# Occurrences of post-traumatic stress disorder, anxiety, depression, and burnout syndrome in ICU staff workers after two-year of the COVID-19 pandemic: the international PSY-CO in ICU study

**DOI:** 10.1186/s12991-023-00488-5

**Published:** 2024-01-03

**Authors:** Claire Roger, Lowel Ling, Mélissa Petrier, Loubna Elotmani, Enora Atchade, Bernard Allaouchiche, Frédéric Aubrun, Jean-Michel Constantin, Claire Dahyot-Fizelier, Nathalie Delhaye, Hervé Dupont, Marc-Olivier Fischer, Marc Garnier, Etienne Gayat, Carole Ichai, Samir Jaber, Jérome Morel, Benoit Plaud, Thomas Rimmelé, Sylvaine Robin, Renee Saba, Gavin M. Joynt, Jean-Yves Lefrant, Pascale Fabbro-Peray, Jeffrey Lipman, Ismael Conejero, Kevin Laupland

**Affiliations:** 1 Division of Anesthesia Critical Care, Pain and Emergency Medicine, Nimes University Hospital, UR‑UM103 IMAGINE, University of Montpellier, Montpellier, France; 2 Department of Anaesthesia and Intensive Care, The Chinese University of Hong Kong, Hong Kong, SAR China; 3 Department of Biostatistics, Epidemiology, Public Health and Innovation in Methodology (BESPIM), CHU Nimes, IDESP, INSERM, University of Montpellier, Nîmes, France; 4 Biostatistics Department, Centre Hospitalier Annecy Genevois, Annecy, France; 5 Department of Anesthesiology and Critical Care Medicine, Assistance Publique-Hôpitaux de Paris (AP-HP), Bichat-Claude Bernard Hospital, Paris, France; 6 Service d’Anesthésie Réanimation, Hospices Civils de Lyon, Pierre-Bénite, France; 7 Department of Anesthesiology and Critical Care, Hôpital de la Croix Rousse, 69317 Lyon, France; 8 Department of Anesthesiology, Critical Care and Perioperative Medicine, Sorbonne Université, GRC 29, AP-HP, DMU DREAM, Hôpital Pitié-Salpetrière, Paris, France; 9 Department of Anesthesia, Intensive Care and Perioperative Medicine, University Hospital of Poitiers, Poitiers, France; 10 INSERM U1070, Pharmacologie des Agents Anti-Infectieux, Poitiers, France; 11 Department of Anesthesiology and Critical Care Medicine, Hôpital Européen Georges Pompidou, AP-HP, Paris, France; 12 Anesthesiology and Critical Care Medicine, Centre Hospitalier Universitaire d’Amiens Picardie, Amiens, France; 13 Normandy University, UNICAEN, CHU de Caen Normandie, Ecole Doctorale NBISE 497, Service d’Anesthésie Réanimation, Caen, France; 14 Institut Aquitain du Coeur, Clinique Saint Augustin, Elsan, 114 Avenue d’Arès, 33074 Bordeaux Cedex, France; 15 Département Médico-Universitaire DREAM, Sorbonne Université, GRC 29, AP-HP, Service d’Anesthésie-Réanimation et Médecine Péri-Opératoire Rive Droite Tenon-Saint Antoine, Paris, France; 16 CHU de Clermont-Ferrand, Université Clermont Auvergne, Service d’Anesthésie-Réanimation et Médecine Périopératoire, 58 Rue Montalembert, 63000 Clermont-Ferrand, France; 17 Department of Anesthesiology and Critical Care, APHP. Nord, DMU Parabol, Université de Paris, Paris, France; 18 UMR-S 942 “MASCOT,” Inserm, Paris, France; 19 Département Anesthésie-Réanimation, Université Côte d’Azur, Centre Hospitalier Universitaire de Nice, Nice, France; 20 Department of Anaesthesia and Intensive Care Unit, Regional University Hospital of Montpellier, St-Eloi Hospital, University of Montpellier, PhyMedExp, INSERM U1046, CNRS UMR, 9214 Montpellier Cedex 5, France; 21 Surgical ICU, Saint-Etienne University Hospital, Saint-Etienne, France; 22 Jacques Lisfranc Medical School, Saint-Etienne University, Saint-Etienne, France; 23 Université Paris Cité, AP-HP. Nord, Hôpital Saint-Louis, DMU PARABOL, Service d’Anesthésie-Réanimation-CTB, 75010 Paris, France; 24 Claude Bernard Lyon 1, Centre Lyonnais d’Enseignement par la Simulation en Santé (CLESS), Lyon, France; 25 EA 7426, PI3 (Pathophysiology of Injury-Induced Immunosuppression), Claude Bernard University Lyon 1-Biomérieux-Hospices Civils de Lyon, Lyon, France; 26 Service d’Anesthésie-Réanimation, Hôpital Edouard Herriot, Hospices Civils de Lyon, Lyon, France; 27 Department of Anesthesia and Critical Care, France Université Grenoble Alpes, CHU Grenoble Alpes, Grenoble, France; 28 Department of Intensive Care Services, Royal Brisbane and Women’s Hospital, Brisbane, QLD Australia; 29 UQ Centre for Clinical Research, The University of Queensland, Brisbane, QLD 4029 Australia; 30 Department of Psychiatry, Nimes University Hospital, Nimes, France; 31 Laboratory of Biochemistry and Molecular Biology, Nimes University Hospital, University of Montpellier, Nimes, France; 32 Institut de Génomique Fonctionnelle, University of Montpellier, CNRS-INSERM, Montpellier, France; 33 Queensland University of Technology (QUT), Brisbane, QLD Australia

**Keywords:** Intensive care unit, ICU staff worker, Post-traumatic stress disorder, Anxiety, Depression, Burnout syndrome

## Abstract

**Purpose:**

The present study aimed at assessing the prevalences of post-traumatic stress disorder (PTSD) (main objective), anxiety, depression, and burnout syndrome (BOS) and their associated factors in intensive care unit (ICU) staff workers in the second year of the COVID-19 pandemic.

**Materials and methods:**

An international cross-sectional multicenter ICU-based online survey was carried out among the ICU staff workers in 20 ICUs across 3 continents. ICUs staff workers (both caregivers and non-caregivers) were invited to complete PCL-5, HADS, and MBI questionnaires for assessing PTSD, anxiety, depression, and the different components of BOS, respectively. A personal questionnaire was used to isolate independent associated factors with these disorders.

**Results:**

PCL-5, HADS, and MBI questionnaires were completed by 585, 570, and 539 responders, respectively (525 completed all questionnaires). PTSD was diagnosed in 98/585 responders (16.8%). Changing familial environment, being a non-caregiver staff worker, having not being involved in a COVID-19 patient admission, having not been provided with COVID-19-related information were associated with PTSD. Anxiety was reported in 130/570 responders (22.8%). Working in a public hospital, being a woman, being financially impacted, being a non-clinical healthcare staff member, having no theoretical or practical training on individual preventive measures, and fear of managing COVID-19 patients were associated with anxiety. Depression was reported in 50/570 responders (8.8%). Comorbidity at risk of severe COVID-19, working in a public hospital, looking after a child, being a non-caregiver staff member, having no information, and a request for moving from the unit were associated with depression. Having received no information and no adequate training for COVID-19 patient management were associated with all 3 dimensions of BOS.

**Conclusion:**

The present study confirmed that ICU staff workers, whether they treated COVID-19 patients or not, have a substantial prevalence of psychological disorders.

**Supplementary Information:**

The online version contains supplementary material available at 10.1186/s12991-023-00488-5.

## Introduction

In December 2019, the coronavirus SARS-CoV-2 resulted in a worldwide outbreak of respiratory illness termed coronavirus disease 2019 (COVID-19), with clinical presentation ranging from asymptomatic disease to severe progressive pneumonia with multiorgan failure. Over 6,537,636 worldwide patients have died (October 12, 2022) [1–3], and although overall mortality is around 3%, the mortality rate of patients admitted to the intensive care unit (ICU) ranges from 20% to more than 60% [1, 3–7]). With few substantially disease modifying antiviral SARS-CoV-2 therapeutic agents, the current therapeutic strategy is based largely on symptomatic treatment and the prevention of transmission [8].

The COVID-19 pandemic presented with different intensities between countries. Therefore, some countries tried to fight and/or delay the start of the pandemic to reduce the peak infection rates of the disease. These actions aimed at reducing the overall pressure on national healthcare systems and was intended to decrease the COVID-19 mortality rate [9, 10].

Based on the experience of previous pandemics, countries reacted by applying different transmission prevention strategies to prevent or delay the spread of the disease [9–11]. Therefore, measures such as border closure, school closure, restricting social gatherings (even shutdown of workplaces), limiting population movements, and lockdowns at the scale of cities or regions were put into action. In public hospitals, several measures were implemented to concentrate care resources on the potential wave of admissions of patients with severe forms of COVID-19. For this reason, the number of available beds in the ICU was frequently increased by up to two-fold [12, 13], and scheduled non-emergency surgical procedures were canceled. Frequently underutilized health care professionals (physicians such as anesthesiologists, and nurses of other units) were transferred to ICUs, and those of less busy units were transferred to busier ones.

All these measures lead to major daily-life changes that could be stressful to individuals. In the general population, it has been well documented that quarantine or confinement, or isolation may lead to the occurrence of post-traumatic stress disorder (PTSD) in about 30% of the exposed population [14]. Importantly, high levels of depressive symptoms have been reported in up to 9% of hospital staff [15]. Numerous symptoms, such as emotional disturbance, depression, stress, low mood, irritability, insomnia, and post-traumatic stress symptoms have been reported after quarantine or isolation [14].

In the ICU setting, it has been shown that the COVID-19 pandemic led to psychological consequences on caregivers. During the second wave in France (autumn 2020), Azoulay et al. reported symptoms of anxiety, depression, post-traumatic stress disorder, and burnout in 60.0%, 36.1%, 28.4, and 45.1%, respectively, in 845 health care providers (66% nursing staff, 32% medical staff, 2% other professionals [16]). However, because the pandemic has continued over a prolonged period, with potentially different impacts on the population and healthcare systems, and varying in intensity according to the vaccination rate, the present study aimed at assessing the occurrence of PTSD, anxiety, depression, and burnout syndrome (BOS) in ICU staff workers in Australia (Queensland), France and Hong Kong after the first year of the COVID-19 pandemic. The primary objective was to assess the prevalence of PTSD in ICU staff workers. The secondary objectives were to identify potential associated factors to the occurrence of PTSD and to assess the prevalence of anxiety, depression, BOS, and their related associated factors in the same cohort.

## Material and methods

### Design

An international cross-sectional multicenter (20 centers) ICU-based online survey was carried out among ICU staff workers in Australia, France, and Hong Kong.

According to French law, this study does not involve patients and is considered a quality-of-care assessment [17]. Therefore, the Institutional Review Board of the Nîmes University Hospital (# 20.05.08) and of the French Society of Anesthesia and Critical Care (IRB 00010254-2020-148) gave their approvals. This study was registered on ClinicalTrial.gov (NCT04511780 first posted on August 13, 2020) before the inclusion of the first participant. In Australia and Hong Kong (SBRE (226-20)), the local ethics committees of each institution gave study approval.

Around the time of the survey administration, in Hong Kong and France there were significant numbers of COVID related admissions to the ICUs, whereas at Royal Brisbane and Women’s Hospital in Brisbane, Australia, COVID-19-related ICU admissions occurred post survey only.

The survey included 5 different questionnaires:

1. The center demographic questionnaire that focused on the nature and organization of the ICU:
  Type of hospital;
  Number of beds in 2020;
  Different categories of staff;
  Number of COVID-19 patients admitted to the unit;
  Alteration in ICU organization during the COVID-19 pandemic (increase in staff, additional beds, educational program for the staff, psychological support);
  Numbers of death among COVID-19 patients.
2. The individual demographic questionnaire that collected personal information:
  Personal socio-demographic data and their changes during the pandemic;
  Professional characteristics (job title, experience), their experience during the COVID-19 pandemic (feeling, family, and professional relationships);
3. Validated questionnaires for assessing PTSD (PCL-5) [18]
4. Hospital Anxiety Depression Scale (HADS) for assessing symptoms of anxiety and depression [19]
5. Maslach Burnout Inventory Human Services Survey for Medical Personnel (MBI-HSS-MP) for assessing BOS [20, 21].

### Study population

The principal investigators contacted ICUs in Australia, France and Hong Kong to participate. After center approval, all ICU staff workers (caregivers in contact with patients and non-caregivers) could participate in the present study. After having had the ability to read an information note about the study, responding to the questionnaire was considered to imply informed consent.

The inclusion criteria were caregiver and non-caregiver staff working in the ICU during the COVID-19 outbreak and consent to complete the questionnaire. The recruitment was performed between February 25th, 2021 and June 8th, 2022.

The non-inclusion criteria were participation refusal and non-response to the questionnaire. Partially completed questionnaires were excluded.

### Outcomes

The primary outcome was the prevalence of PTSD (defined by a PCL-5 score ≥ 32) and its 95% confident interval (95% CI).

The secondary outcomes were to identify potential associated factors with occurrence of PTSD and to the prevalences of anxiety and depression according to the HADS questionnaire, and burnout assessed by the MBI-HSS (MP) self-questionnaire.

Anxiety and depression were separately assessed by the HADS questionnaire according to the following rules:

- 0 to 7: absence of disorder;
- 8 to 10: suspected disorder;
- 11 to 21: proven disorder.

Burnout syndrome was assessed by the MBI-HSS (MP) in its 3 specific sub-scales allowing for the evaluation of emotional exhaustion, depersonalization, and personal accomplishment dimensions, respectively. However, many controversies remain unsolved for the global MBI assessment: [20, 22]

1. Personal accomplishment is not always taken into account in the global MBI score;
2. In each subscale, the different thresholds are challenged.

Thus, we have analyzed the 3 sub-scores both separately and continuously.

### Statistical analysis

The primary objective, i.e., to evaluate the prevalence of PTSD, was measured with the PCL-5 score and classified as probable PTSD versus no PTSD with PCL-5 scores of ≥ 32 versus < 32 with 95% confidence intervals (95% CI), respectively. The prevalence of PTSD was estimated in the total sample and in each country.

The associated factors with PTSD were searched as secondary objectives. For this purpose, we selected variables with univariate logistic regression to reduce the dimensionality of the model (relaxed alpha = 0.2) and then applied a multivariate logistic regression with backward selection (alpha = 0.05). First, the univariate analysis compared the dichotomous/categorical/nominal variables (expressed as numbers and percentages) according to PTSD occurrence by the chi-square test or the Fisher exact test when necessary. The links between the explanatory variables and PTSD variables were expressed by the odds ratios and their 95% CI by the Wald method. Covariates with a *p*-value ≤ 0.20 in the univariate analysis were pre-selected to perform a multivariate analysis and a backward selection strategy at the 5% threshold was applied. Adjusted Odds Ratio (AOR) was provided with 95% CI. Importantly, the prevalence of PTSD was assessed in all completed PCL-5 (*n* = 585) whereas the associated factors were searched in participants who completed PCL-5 AND personal life questionnaires (*n* = 525).

For the other secondary objectives, the same analysis strategy was applied to evaluate the prevalence of anxiety, depression on one hand, and the factors associated with these disorders on the other (using the same method used for PTSD and associated factors). A polytomous logistic regression with a proportional-odds cumulative logit model was used to search for factors associated with anxiety and depression classified in a 3-level ordinal variable. The scores of the emotional exhaustion, depersonalization, and personal accomplishment subscales were expressed as mean, standard deviation (SD), median and interquartile range (IQR). The associated factors to the 3 sub-scores were assessed with a multiple linear regression model. The same variable selection strategy was used for the previous models. Pearson correlation coefficients between PTSD, anxiety, depression, emotional exhaustion, depersonalization, and personal accomplishment scores are provided with their 95% CI. All statistical analyses used SAS statistical software, version 9.4 (SAS Institute Inc).

## Results

**Fig. 1:**
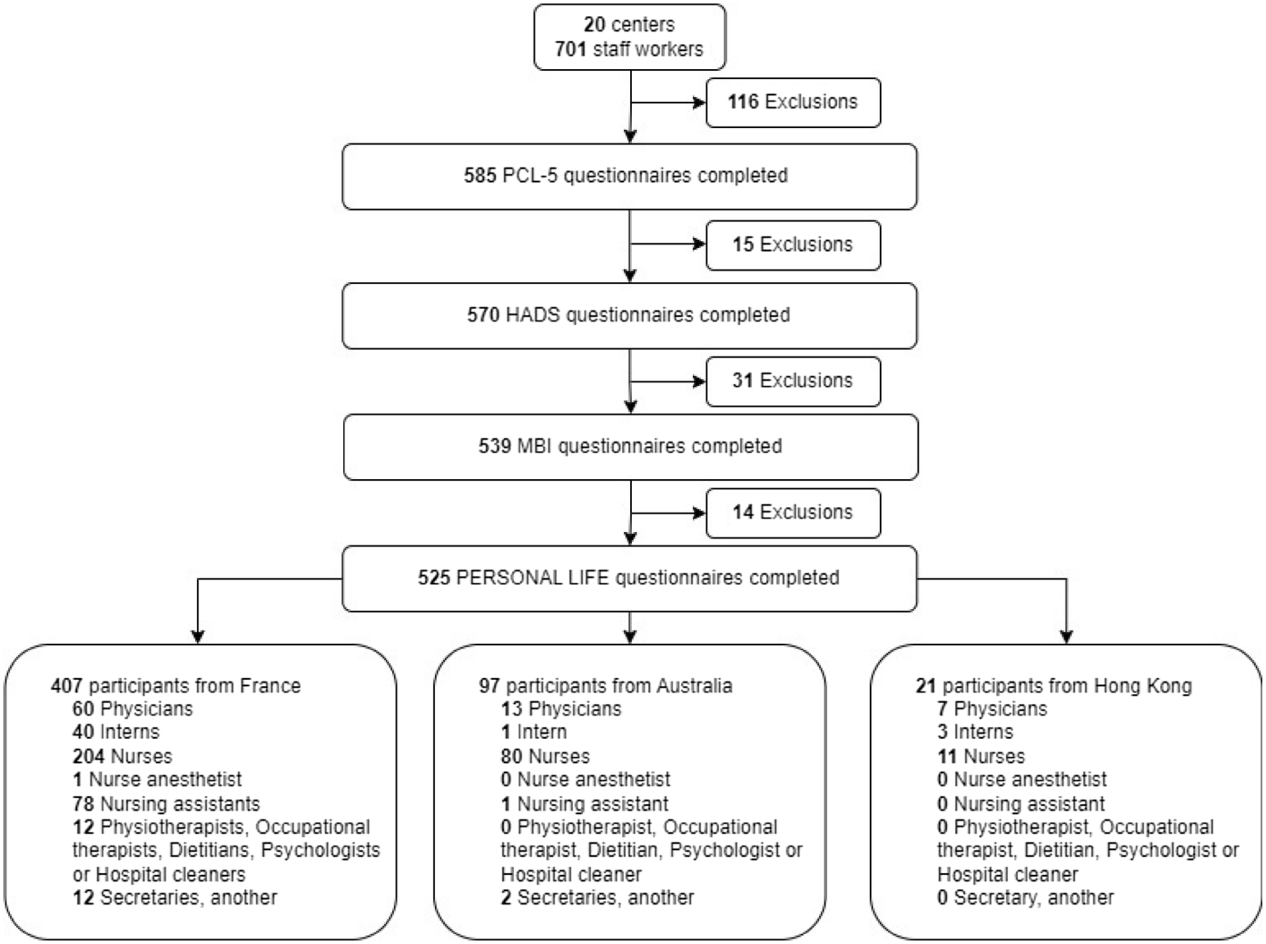
Flowchart illustrating the selection and participation of the study

The flowchart is shown in Fig. 1. Among 701 responders (in 20 different centers), 585, 570, and 539 completed PCL-5, HADS, and MBI questionnaires, respectively. All questionnaires were completed by 525 responders (511 caregivers and 14 non-caregivers).

### PTSD prevalence

A PCL-5 score ≥ 32 was reported in 98 out of 585 responders (prevalence = 16.8%, 95% CI [13.7–19.8%]) with significant difference between countries: France (prevalence = 74/448, 16.5% 95% CI [13.1–20.0%]), Australia (prevalence = 16/111, 14.4% 95% CI [7.9–21.0%]) and Hong Kong (prevalence = 8/26, 30.8% 95% CI [13.0–48.5%].

According to the multivariate analysis (including 525 participants who fully completed PCL-5 and personal life questionnaires), 5 factors were associated with greater frequency of PTSD (Table 1): changing in the home environment during the COVID-19 pandemic, being a non-caregiver, having no COVID-19 patient admission, and no information on the evolution of the pandemic.

**Table 1:** Associated factors with the presence of PTSD

*N* = 525^*^	PTSD, No^ƚ^. /Total No. (%)	Univariate analysis^ǂ^		Multivariate analysis^*^ (N = 525)	
		OR (95% CI)	*p*-value	AOR (95% CI)^§^	*p*-value
*Factors*					
Type of hospital					
University Hospital	73/470 (15.5)	1 [Reference]	.17	NA**	NA
Public Hospital	13/52 (25.0)	1.8 (0.9–3.6)		NA	NA
Private Hospital	1/3 (33.3)	2.7 (0.2–30.5)		NA	NA
Gender					
Male	19/146 (13.0)	1 [Reference]	.17	NA	NA
Female	68/379 (17.9)	1.5 (0.8–2.5)		NA	NA
Living with a partner					
No	31/147 (21.1)	1 [Reference]	.08	NA	NA
Yes	56/378 (14.8)	0.7 (0.4–1.1)		NA	NA
Changing of residence during the pandemic					
No	67/432 (15.5)	1 [Reference]	**.16**	1 [Reference]	**.03**
Yes	20/93 (21.5)	1.5 (0.9–2.6)		1.9 (1.0–3.3)	
Financially impacted during the pandemic					
No	66/443 (14.9)	1 [Reference]	.01	NA	NA
Yes	21/82 (25.6)	2.0 ( 1.1–3.4)		NA	NA
Occupation					
Caregiver	73/511 (15.5)	1 [Reference]	**.0004**	1 [Reference]	**.0002**
Non-caregiver	8/14 (57.1)	7.3 (2.5–21.6)		8.9 (2.9–27.7)	
Admission of COVID-19 patient					
Yes	7/19 (36.8)	1 [Reference]	**.02**	1 [Reference]	**.01**
No	80/506 (15.8)	3.1 (1.2–8.2)		3.9 (1.4–11.0)	
Theoretical or practical training on individual preventive measures for managing COVID-19 patient					
No	29/134 (21.6)	1 [Reference]	.06	NA	NA
Yes	58/391 (14.8)	0.6 (0.4–1.0)		NA	NA
Regularly information on the evolution of the pandemic					
Yes	55/396 (13.9)	1 [Reference]	**.004**	1 [Reference]	**.001**
No	32/129 (24.8)	2.0 (1.3–3.3)		2.3 (1.4–3.9)	
Sufficient training to manage COVID-19 patient					
No	46/215 (21.4)	1 [Reference]	.01	NA	NA
Yes	30/129 (23.3)	0.6 (0.4–0.9)		NA	NA
Sufficient information for managing COVID-19 patient					
Yes	51/346 (14.7)	1 [Reference]	.11	NA	NA
No	36/179 (20.1)	1.5 ( 0.9–2.3)		NA	NA
Sufficient personal protective equipment					
Yes	51/345 (14.8)	1 [Reference]	.12	NA	NA
No	36/180 (20.0)	1.4 (0.9–2.3)		NA	NA
Refusal to admit patients to the ICU even with available beds according to predefined criteria					
No	43/295 (14.6)	1 [Reference]	.16	NA	NA
Yes	44/230 (19.1)	1.4 (0.9–2.2)		NA	NA
Management of COVID-19 patient				NA	
Yes	75/486 (15.4)	1 [Reference]	.01	NA	NA
No	12/39 (30.8)	2.4 (1.2–5.0)		NA	NA
Agree to manage COVID-19 patient					
Yes	64/416 (15.4)	1 [Reference]	.10	NA	NA
No	23/109 (21.1)	1.9 (1.2–3.0)		NA	NA
Fear for managing COVID-19 patient					
No	34/249 (13.7)	1 [Reference]	.08	NA	NA
Yes	53/276 (19.2)	1.5 (0.9–2.4)		NA	NA
Comorbidity at risk of severe COVID-19					
No	62/437 (14.2)	1 [Reference]	**.001**	1 [Reference]	**.0004**
Yes	25/88 (28.4)	2.4 (1.4–4.1)		2.8 (1.6–4.9)	
Close family member contaminated with COVID-19					
No	39/269 (14.5)	1 [Reference]	.19	NA	NA
Yes	48/256 (18.8)	1.4 (0.9–2.2)		NA	NA

^*^According to the order of appearance of the survey forms, an imbalance in the completion rate was noted between the first questionnaire (Personal life questionnaire) and the last form (Personal and Professional questionnaire used to research the factors associated with the psychological disorders studied) (higher completion rate for the first questionnaire). To evaluate the prevalence associated with psychological disorders, all the answers filled in for each scale of evaluation of the latter were taken into account, although the questionnaire was not completed in full. For this reason, a difference in the numbers analyzed (between those for the prevalence of post-traumatic stress, anxiety, and depression and those for the analysis of associated factors) is observed (see Fig. 1). The search for factors associated with the occurrence of psychological disorders was carried out on 525 people (those who completed all the survey forms)

^ƚ^Number of observations / total number of observations

^ǂ^The results presented correspond to the pre-selection of variables at *p*-value < 20%. The second selection of variables was made at the 5% threshold and then integrated into the multivariate model

^§^Adjusted odd ratio with a 95% confidence interval

^**^Not applicable

PCL-5 score was highly correlated with anxiety (*r* = 0.73, 95% CI [0.69–0.77], *p* < 0.0001), depression (*r* = 0.73, 95% CI [0.69–0.77], *p* < 0.0001) and emotional exhaustion (*r* = 0.70, 95% CI [0.62–0.71], *p* < 0.0001) scores (Additional file 1: Table S1).

### Anxiety

A positive anxiety disorder (HADS score between 11 and 21) was reported in 130 out of 570 responders (prevalence = 22.8%, 95% CI [19.4–26.3%]) with no difference between countries: France (prevalence = 98/438, 22.4% 95% CI [18.5–26.3%]), Australia (prevalence = 26/108, 24.1% 95% CI [16.0–32.1%]) and Hong Kong (prevalence = 6/24, 25.0% 95% CI [7.7–42.3%]).

According to the multivariate analysis (including 525 participants who fully completed HADS and personal life questionnaires), working in a public hospital, being a woman, being financially impacted during the pandemic, being a non-caregiver, having no theoretical or practical training on individual preventive measures, and fear of managing COVID-19 patients were associated with a greater frequency of proven anxiety disorder (Table 2).

### Depression

A positive depressive disorder (HADS score between 11 and 21) was reported in 50 out of 570 responders (prevalence = 8.8%, 95% CI [6.5–11.1%]) with significant difference between countries: France (prevalence = 40/438, 9.1% 95% CI [6.4–11.8%]), Australia (prevalence = 9/108, 8.3% 95% CI [3.1–13.6%]) and Hong Kong (prevalence = 1/24, 4.2% 95% CI [0.0–12.2%]).

According to the multivariate analysis (including 525 participants who fully completed HADS and personal life questionnaires), comorbidity at risk of severe COVID-19, working in a public hospital, looking after a child, being a non-caregiver, having no information on the evolution of the pandemic, having requested a change of unit for not working in a COVID unit were associated with a greater occurrence of proven depressive disorder (Table 2).

**Table 2:** Associated factors with the anxiety and the depression

*N* = 525^*^	Proven anxiety, No^ƚ^. /Total No. (%)	Univariate analysis^ǂ^		Multivariate analysis^*^ (*N* = 525)	
		OR (95% CI)	*p*-value	AOR (95% CI)^§^	*p-*value
Anxiety					
*Factors*					
Type of hospital					
University hospital	94/470 (20.0)	1 [Reference]	**.001**	1 [Reference]	**.01**
Public hospital	20/52 (38.5)	2.6 (1.5–4.4)		2.3 (1.3–3.9)	
Private hospital	2/3 (66.7)	6.0 (0.6–56.9)		2.8 (0.2–30.6)	
Gender					
Male	24/146 (16.4)	1 [Reference]	** < .0001**	1 [Reference]	**.0008**
Female	92/379 (24.3)	2.5 (1.7–3.8)		2.1 (1.4–3.2)	
Type of housing					
House	65/251 (25.9)	1 [Reference]	.003	NA^**^	NA
Apartment	51/274 (18.6)	0.7 (0.5–1.0)		NA	NA
Housing with an exterior					
Yes	91/385 (23.6)	1 [Reference]	.05	NA	NA
No	25/140 (17.9)	0.7 (0.7–1.0)		NA	NA
Financially impacted during the pandemic					
No	91/443 (20.5)	1 [Reference]	**.01**	1 [Reference]	**.02**
Yes	25/82 (30.5)	1.8 (1.1–2.8)		1.7 (1.1–2.7)	
Occupation					
Caregiver	108/511 (21.1)	1 [Reference]	**.002**	1 [Reference]	**.01**
Non-caregiver	8/14 (57.1)	5.0 (1.8–14.2)		3.9 (1.3–11.3)	
Theoretical or practical training on individual preventive measures for managing COVID-19 patient					
Yes	76/391 (19.4)	1 [Reference]	**.01**	1 [Reference]	**.04**
No	40/134 (29.9)	1.6 (1.1–2.4)		1.5 (1.0–2.2)	
Sufficient training to manage COVID-19 patient					
Yes	58/310 (18.7)	1 [Reference]	.05	NA	NA
No	58/215 (27.0)	1.4 (1.0–2.0)		NA	NA
Sufficient information for managing COVID-19 patient					
Yes	64/346 (18.5)	1 [Reference]	.008	NA	NA
No	52/179 (29.1)	1.6 (1.1–2.3)		NA	NA
Refusal to admit patients to the ICU even with available beds according to predefined criteria					
Yes	44/230 (19.1)	1 [Reference]	.02	NA	NA
No	72/295 (24.4)	1.5 (1.1–2.1)		NA	NA
Management of COVID-19 patient					
Yes	101/486 (20.8)	1 [Reference]	< .0001	NA	NA
No	15/39 (38.5)	2.5 (1.4–4.6)		NA	NA
Agree to manage COVID-19 patient					
Yes	83/416 (20.0)	1 [Reference]	.01	NA	NA
No	33/109 (30.3)	1.6 (1.1–2.4)		NA	NA
Fear for managing COVID-19 patient					
No	47/249 (18.9)	1 [Reference]	**.002**	1 [Reference]	**.02**
Yes	69 /276 (25.0)	1.7 (1.2–2.4)		1.5 (1.0–2.1)	
Comorbidity at risk of severe COVID-19					
No	92/437 (21.1)	1 [Reference]	.03	NA	NA
Yes	24/88 (27.3)	1.6 (1.0–2.5)		NA	NA

*N* = 525^*^	Proven depression, No. /Total No. (%)	Univariate analysis^ǂ^		Multivariate analysis^*^ (*N* = 525)	
		OR (95% CI)	*p*-value	AOR (95% CI)	*p*-value
Depression					
*Factors*					
Type of hospital					
University hospital	36/470 (7.7)	1 [Reference]	**.01**	1 [Reference]	**.03**
Public hospital	7/52 (13.5)	2.4 (1.3–4.3)		2.2 (1.2–4.1)	
Private hospital	0/3 (0.0)	3.7 (0.4–32.0)		2.9 (0.3–28.3)	
Gender					
Male	8/146 (5.5)	1 [Reference]	.02	NA	NA
Female	32/379 (9.2)	1.7 (1.1–2.8)		NA	NA
Type of housing					
House	20/251 (8.0)	1 [Reference]	.06	NA	NA
Apartment	23/274 (8.4)	0.7 (0.5–1.0)		NA	NA
Housing with an exterior					
Yes	32/385 (8.3)	1 [Reference]	.01	NA	NA
No	11/140 (7.9)	0.5 (0.3–0.9)		NA	NA
Looking after a child					
No child to support/not concerned/no	27/395 (6.8)	1 [Reference]	**.02**	1 [Reference]	**.01**
Yes	16/130 (12.3)	1.7 (1.1–2.6)		1.8 (1.1–2.8)	
Financially impacted during the pandemic					
No	36/443 (8.1)	1 [Reference]	.13	NA	NA
Yes	7/82 (8.5)	1.5 (0.9–2.5)		NA	NA
Occupation					
Caregiver	38/511 (7.4)	1 [Reference]	**.0003**	1 [Reference]	**.0001**
Non-caregiver	5/14 (35.7)	6.4 (2.4–17.2)		8.0 (2.8–22.7)	
Admission of COVID-19 patient					
Yes	40/506 (7.9)	1 [Reference]	.07	NA	NA
No	3/19 (15.8)	2.3 (0.9–5.7)		NA	NA
Theoretical or practical training on individual preventive measures for managing COVID-19 patient					
Yes	30/391 (7.8)	1 [Reference]	.01	NA	NA
No	13/134 (9.7)	1.7 (1.1–2.6)		NA	NA
Hands-on training in the management of a COVID-19 patient					
Yes	21/291 (7.2)	1 [Reference]	.01	NA	NA
No	22/234 (9.4)	1.7 (1.1–2.5)		NA	NA
Regularly information on the evolution of the pandemic					
Yes	34/396 (8.6)	1 [Reference]	**.01**	1 [Reference]	**.04**
No	9/129 (7.0)	1.7 (1.1–2.7)		1.6 (1.0–2.5)	
Sufficient training to manage COVID-19 patient					
Yes	22/310 (7.1)	1 [Reference]	**.0003**	1 [Reference]	**.004**
No	21/215 (9.8)	2.1 (1.4–3.1)		1.8 (1.2–2.8)	
Sufficient information for managing COVID-19 patient					
Yes	22/346 (6.4)	1 [Reference]	.001	NA	NA
No	21/179 (11.7)	2.0 (1.3–3.0)		NA	NA
Sufficient personal protective equipment					
Yes	24/345 (7.0)	1 [Reference]	.04	NA	NA
No	19/180 (10.6)	1.5 (1.0–2.3)		NA	NA
Management of COVID-19 patient					
Yes	35/486 (7.2)	1 [Reference]	.0007	NA	NA
No	8/39 (20.5)	3.0 (1.6–5.7)		NA	NA
Agree to manage COVID-19 patient					
Yes	33/416 (7.9)	1 [Reference]	.13	NA	NA
No	10/109 (9.2)	1.4 (0.9–2.3)		NA	NA
Fear for managing COVID-19 patient					
No	18/249 (7.2)	1 [Reference]	.02	NA	NA
Yes	25/276 (9.1)	1.6 (1.1–2.4)		NA	NA
Request for moving from the unit					
No	39/503 (7.8)	1 [Reference]	**.01**	1 [Reference]	**.02**
Yes	4/22 (18.2)	2.7 (1.2–6.2)		2.6 (1.1–6.3)	
Comorbidity at risk of severe COVID-19					
No	29/437 (6.6)	1 [Reference]	**.0004**	1 [Reference]	**.001**
Yes	14/88 (15.9)	2.4 (1.5–3.8)		2.2 (1.4–3.7)	

^*^According to the order of appearance of the survey forms, an imbalance in the completion rate was noted between the first questionnaire (Personal life questionnaire) and the last form (Personal and Professional questionnaire used to research the factors associated with the psychological disorders studied) (higher completion rate for the first questionnaire). To evaluate the prevalence associated with psychological disorders, all the answers filled in for each scale of evaluation of the latter were taken into account, although the questionnaire was not completed in full. For this reason, a difference in the numbers analyzed (between those for the prevalence of post-traumatic stress, anxiety, and depression and those for the analysis of associated factors) is observed (see Fig. 1). The search for factors associated with the occurrence of psychological disorders was carried out on 525 people (those who completed all the survey forms)

^ƚ^ Number of observations / Total number of observations

^ǂ^ The results presented correspond to the pre-selection of variables at *p*-value < 20%. The second selection of variables was made at the 5% threshold and then integrated into the multivariate model

^§^ Adjusted odd ratio with a 95% confidence interval

^**^ Not Applicable

### Sub-scores of burnout

#### Emotional exhaustion

The emotional exhaustion score in the total sample was 23.5 ± 13.7.

According to the multivariate analysis (including 525 participants who fully completed MBI and personal life questionnaires), usually living alone, being a non-caregiver, having no information on the evolution of the pandemic, not being adequately trained to manage a COVID-19 patient, not having accepted managing COVID-19 patients, and fear of managing a COVID-19 patient were independently associated with greater emotional exhaustion (Table 3).

**Table 3:** Associated factors with the emotional exhaustion, depersonalization, and personal accomplishment scores

*N* = 525^*^	Score mean (SD)^ƚƚ^	Median (IQR)^ǂǂ^	Univariate analysis^ǂ^		Multivariate analysis^*^ (*N* = 525)	
			*β*^§§^	*p*-value	*β*^§§^	*p*-value
Emotional exhaustion						
*Factors*						
Type of hospital						
University hospital (*n* = 470)	22.9 (13.7)	22.0 (23.0)	1 [Reference]	.03	NA^**^	NA
Public hospital (*n* = 52)	27.3 (13.1)	25.0 (25.0)	4.5		NA	NA
Private hospital (*n* = 3)	36.0 (15.6)	44.0 (28.0)	12.8			
Gender						
Male (*n* = 146)	20.9 (14.0)	19.0 (22.0)	1 [Reference]	.002	NA	NA
Female (*n* = 379)	24.5 (13.5)	24.0 (23.0)	4.0		NA	NA
Age (years)						
< 30 (*n* = 171)	25.2 (13.5)	25.0 (24.0)	1 [Reference]	.09	NA	NA
30–39 (*n* = 187)	23.0 (13.7)	21.0 (22.0)	- 1.9		NA	NA
40–49 (*n* = 101)	23.1 (14.5)	21.0 (25.0)	- 2.2		NA	NA
≥ 50 (*n* = 66)	20.8 (13.0)	19.5 (22.0)	- 4.9		NA	NA
Usually, live alone						
No (*n* = 400)	22.7 (13.7)	20.0 (23.0)	1 [Reference]	**.04**	1 [Reference]	**.03**
Yes (*n* = 125)	26.1 (13.4)	29.0 (23.0)	2.9		2.9	
Living with a partner						
No (*n* = 147)	25.2 (13.9)	25.0 (25.0)	1 [Reference]	.10	NA	NA
Yes (*n* = 378)	22.8 (13.6)	26.0 (24.5)	- 2.1		NA	NA
Financially impacted during the pandemic						
No (*n* = 443)	22.8 (13.6)	21.0 (23.0)	1 [Reference]	.007	NA	NA
Yes (*n* = 82)	27.3 (13.5)	29.0 (21.0)	4.3		NA	NA
Occupation						
Caregiver (*n* = 511)	23.2 (13.6)	22.0 (22.0)	1 [Reference]	**.02**	1 [Reference]	**.02**
Non-caregiver (*n* = 14)	32.1 (15.2)	37.0 (22.0)	8.2		7.8	
Theoretical or practical training on individual preventive measures for managing COVID-19 patient						
Yes (*n* = 391)	22.7 (13.3)	21.0 (22.0)	1 [Reference]	.01	NA	NA
No (*n* = 134)	25.7 (14.7)	25.0 (26.0)	3.5		NA	NA
Hands-on training in the management of a COVID-19 patient						
Yes (*n* = 291)	22.8 (13.3)	21.0 (21.0)	1 [Reference]	.01	NA	NA NA
No (*n* = 234)	24.4 (14.2)	23.5 (25.0)	3.0		NA	
Regularly information on the evolution of the pandemic						
Yes (*n* = 396)	22.0 (13.3)	20.0 (21.0)	1 [Reference]	** < .0001**	1 [Reference]	**.0002**
No (*n* = 129)	28.1 (14.1)	30.0 (25.0)	5.8		5.0	
Sufficient training to manage COVID-19 patient						
Yes (*n* = 310)	21.7 (13.2)	20.0 (21.0)	1 [Reference]	**.0001**	1 [Reference]	**.01**
No (*n* = 215)	26.0 (14.0)	27.0 (24.0)	4.6		3.0	
Sufficient information for managing COVID-19 patient						
Yes (*n* = 346)	21.8 (13.5)	20.0 (22.0)	1 [Reference]	< .0001	NA	NA
No (*n* = 179)	26.7 (13.6)	27.0 (24.0)	5.0		NA	NA
Sufficient personal protective equipment						
Yes (*n* = 345)	22.3 (13.8)	20.0 (23.0)	1 [Reference]	.01	NA	NA
No (*n* = 180)	25.8 (13.3)	26.0 (22.0)	3.3		NA	NA
Management of COVID-19 patient						
Yes (*n* = 486)	23.2 (13.6)	22.0 (22.0)	1 [Reference]	.12	NA	NA
No (*n* = 39)	27.0 (14.8)	29.0 (25.0)	3.5		NA	NA
Agree to manage COVID-19 patient						
Yes (*n* = 416)	22.3 (13.5)	20.5 (23.0)	1 [Reference]	** < .0001**	1 [Reference]	** < .0001**
No (*n* = 109)	28.0 (13.6)	29.0 (22.0)	8.5		7.1	
Fear of managing COVID-19 patient						
No (*n* = 249)	21.0 (13.4)	18.0 (21.0)	1 [Reference]	** < .0001**	1 [Reference]	** < .0001**
Yes (*n* = 276)	25.8 (13.6)	26.0 (22.0)	5.4		4.5	
Request for moving from the unit						
No (*n* = 503)	23.3 (13.8)	22.0 (24.0)	1 [Reference]	.11	NA	NA
Yes (*n* = 22)	27.2 (11.9)	29.5 (21.0)	4.7		NA	NA
SARS-COV-2 contamination						
No (*n* = 404)	23.1 (13.6)	21.0 (22.5)	1 [Reference]	.13	NA	NA
Yes (*n* = 121)	24.9 (14.0)	27.0 (24.0)	2.1		NA	NA
Comorbidity at risk of severe COVID-19						
No (*n* = 437)	23.0 (13.6)	21.0 (23.0)	1 [Reference]	.13	NA	NA
Yes (*n* = 88)	25.9 (14.1)	26.0 (24.0)	2.4		NA	NA

*N* = 525	Score mean (SD)	Median (IQR)	Univariate analysis		Multivariate analysis (N = 525)	
			*β*	*p*-value	*β*	*p*-value
Depersonalization						
*Factors*						
Age (years)						
< 30 (*n* = 171)	11.0 (6.8)	10.0 (11.0)	1 [Reference]	** < .0001**	1 [Reference]	** < .0001**
30–39 (*n* = 187)	9.5 (7.2)	8.0 (10.0)	- 1.3		- 1.3	
40–49 (*n* = 101)	7.4 (6.6)	5.0 (9.0)	- 3.3		- 3.1	
≥ 50 (*n* = 66)	5.8 (5.6)	3.5 (8.0)	- 5.0		- 4.4	
Type of housing						
House (*n* = 251)	8.1 (6.6)	6.0 (9.0)	1 [Reference]	.07	NA	NA
Apartment (*n* = 274)	10.0 (7.2)	8.0 (11.0)	1.2		NA	NA
Looking after a child						
No child to support/Not concerned/No (*n* = 395)	9.4 (7.2)	8.0 (12.0)	1 [Reference]	.14	NA	NA
Yes (*n* = 130)	8.1 (6.2)	6.0 (8.0)	- 1.0		NA	NA
Living separately from his/her partner during the pandemic						
Not concerned/No (*n* = 463)	8.9 (6.8)	7.0 (10.0)	1 [Reference]	.06	NA	NA
Yes (*n* = 62)	10.8 (7.8)	9.0 (14.0)	1.7		NA	NA
Occupation						
Caregiver (*n* = 511)	9.0 (7.0)	8.0 (11.0)	1 [Reference]	.12	NA	NA
Non-caregiver (*n* = 14)	12.1 (7.7)	11.5 (15.0)	2.9		NA	NA
SARS-COV-2 contamination						
No (*n* = 404)	8.5 (6.8)	7.0 (10.0)	1 [Reference]	**.004**	1 [Reference]	**.01**
Yes (*n* = 121)	11.0 (7.2)	10.0 (12.0)	2.1		1.7	
Usually, live alone						
No (*n* = 400)	8.6 (6.8)	7.0 (10.0)	1 [Reference]	.03	NA	NA
Yes (*n* = 125)	10.6 (7.5)	9.0 (11.0)	1.5		NA	NA
Hands-on training in the management of a COVID-19 patient						
Yes (*n* = 291)	8.9 (6.7)	8.0 (10.0)	1 [Reference]	.10	NA	NA
No (*n* = 234)	9.4 (7.3)	8.0 (12.0)	1.0		NA	NA
Regularly information on the evolution of the pandemic						
Yes (*n* = 396)	8.4 (6.6)	7.0 (10.0)	1 [Reference]	** < .0001**	1 [Reference]	**.0003**
No (*n* = 129)	11.3 (7.8)	10.0 (13.0)	2.8		2.5	
Sufficient training to manage COVID-19 patient						
Yes (*n* = 310)	8.5 (7.3)	7.0 (10.0)	1 [Reference]	.009	NA	NA
No (*n* = 215)	9.9 (7.3)	9.0 (12.0)	1.6		NA	NA
Sufficient information for managing COVID-19 patient						
Yes (*n* = 346)	8.6 (6.6)	7.0 (10.0)	1 [Reference]	.01	NA	NA
No (*n* = 179)	10.1 (7.6)	8.0 (12.0)	1.6		NA	NA
Sufficient personal protective equipment						
Yes (*n* = 345)	8.6 (6.7)	7.0 (10.0)	1 [Reference]	.16	NA	NA
No (*n* = 180)	10.0 (7.4)	8.5 (12.0)	0.9		NA	NA
Refusal to admit patients to the ICU even with available beds according to predefined criteria						
No (*n* = 295)	8.2 (6.5)	7.0 (9.0)	1 [Reference]	.04	NA	NA
Yes (*n* = 309)	9.7 (7.3)	9.0 (12.0)	1.2		NA	NA
Refusal to admit patients to the ICU because of unavailable beds	6.1 (5.8)	5.0 (7.5)				
No (*n* = 216)	7.4 (6.4)	6.0 (10.0)	1 [Reference]	.007	NA	NA
Yes (*n* = 230)	7.2 (6.6)	5.0 (9.0)	1.7		NA	NA

*N* = 525	Score mean (SD)	Median (IQR)	Univariate analysis		Multivariate analysis (N = 525)	
			*β*	*p*-value	*β*	*p*-value
Personal accomplishment						
*Factors*						
Type of hospital						
University hospital (*n* = 470)	35.7 (7.9)	37.0 (11.0)	1 [Reference]	**.003**	1 [Reference]	**.002**
Public hospital (*n* = 52)	31.7 (7.9)	31.0 (12.5)	- 3.9		- 3.8	
Private hospital (*n* = 3)	35.0 (5.2)	32.0 (9.0)	- 0.6		0.3	
Occupation						
Healthcare staff (*n* = 511)	35.4 (7.9)	36.0 (11.0)	1 [Reference]	.06	NA	NA
Non-healthcare staff (*n* = 14)	31.5 (8.4)	33.0 (10.0)	- 3.9		NA	NA
Usually works in an ICU						
Yes (*n* = 484)	35.4 (7.8)	36.0 (11.0)	1 [Reference]	.20	NA	NA
No (*n* = 41)	33.9 (9.0)	33.0 (15.0)	- 1.6		NA	NA
Theoretical or practical training on individual preventive measures for managing COVID-19 patient						
Yes (*n* = 391)	36.3 (7.4)	37.0 (11.0)	1 [Reference]	** < .0001**	1 [Reference]	** < .0001**
No (*n* = 134)	32.2 (8.6)	34.0 (13.0)	- 4.1		- 3.3	
Hands-on training in the management of COVID-19 patient						
Yes (*n* = 291)	36.6 (7.3)	38.0 (11.0)	1 [Reference]	< .0001	NA	NA
No (*n* = 234)	33.7 (8.4)	35.0 (12.0)	- 2.9		NA	NA
Sufficient training to manage COVID-19 patient						
Yes (*n* = 310)	36.5 (7.4)	38.0 (10.0)	1 [Reference]	< .0001	NA	NA
No (*n* = 215)	33.4 (8.3)	34.0 (12.0)	- 3.1		NA	NA
Sufficient information for managing COVID-19 patient						
Yes (*n* = 346)	36.6 (7.3)	32.0 (13.0)	1 [Reference]	** < .0001**	1 [Reference]	** < .0001**
No (*n* = 179)	32.6 (8.5)	32.0 (11.0)	- 4.0		- 3.1	
Comorbidity at risk of severe COVID-19						
No (*n* = 437)	35.7 (7.7)	37.0 (12.0)	1 [Reference]	**.004**	1 [Reference]	**.01**
Yes (*n* = 88)	33.0 (8.6)	35.0 (10.0)	- 2.6		- 2.1	

^*^According to the order of appearance of the survey forms, an imbalance in the completion rate was noted between the first questionnaire (Personal life questionnaire) and the last form (Personal and Professional questionnaire used to research the factors associated with the psychological disorders studied) (higher completion rate for the first questionnaire). To evaluate the prevalence associated with psychological disorders, all the answers filled in for each scale of evaluation of the latter were taken into account, although the questionnaire was not completed in full. For this reason, a difference in the numbers analyzed (between those for the prevalence of post-traumatic stress, anxiety, and depression and those for the analysis of associated factors) is observed (see Fig. 1). The search for factors associated with the occurrence of psychological disorders was carried out on 525 people (those who completed all the survey forms)

^ƚ^Number of observations / Total number of observations

^ǂ^The results presented correspond to the pre-selection of variables at *p*-value < 20%. The second selection of variables was made at the 5% threshold and then integrated into the multivariate model

^§^Adjusted odd ratio with a 95% confidence interval

^**^Not applicable

^ƚƚ^Standard deviation

^ǂǂ^Interquartile range

^§§^Regression coefficient

#### Depersonalization

The depersonalization score in the total sample was 9.1 ± 7.0.

According to the multivariate analysis, having been infected with SARS-CoV-2 and having no information on the evolution of the pandemic were associated with a higher depersonalization score. An age > 50 years was associated with lower depersonalization (Table 3).

#### Personal accomplishment

The loss of personal accomplishment score in the total sample was 35.3 ± 7.9.

According to the multivariate analysis, comorbidity at risk of severe COVID-19, working in a public hospital, having no theoretical or practical training on individual preventive measures, and insufficient information about the management of COVID-19 patients were associated with lower personal accomplishment (Table 3).

Emotional exhaustion and Depersonalization scores were both correlated (*r* = 0.57, 95% CI [0.51–0.63], *p* < 0.0001), whereas the latter were negatively but less correlated with personal accomplishment (Additional file 1: Table S1). The position and dispersion parameters associated with each score are reported in Additional file 1: Table S1.

## Discussion

In the present study performed in 20 centers in Australia, France, and Hong Kong, 525 ICU staff workers responded to the PCL-5, HADS, and MBI questionnaires. PTSD was present in 16.8% of participants with the highest prevalence in Hong Kong (30.8%). Anxiety and depressive disorders were reported in 22.8 and 8.8% of responders, respectively. The common associated factors with PTSD, anxiety, and depression were being a non-caregiver worker and not having been regularly informed of the COVID-19 progression during the pandemic. Concerning BOS, not having been regularly informed of the COVID-19 progression was associated with higher scores for emotional exhaustion, depersonalization, and the loss of personal accomplishment, respectively.

The present study was performed during the second year of the COVID-19 pandemic in 3 different countries with different impacts of this pandemic, different strategies to prevent contamination, and different population vaccination rates. These factors could explain the different prevalences of PTSD, anxiety, and depression reported in previous studies that were essentially performed in European countries during the first and second waves. The FAMIREA group performed two studies in the first and second waves in 21 and 16 centers involving 845 (70% responders) and 1058 (67% responders healthcare professionals, respectively [16, 23]. The prevalences of PTSD were successively 32.0 and 28.4% with anxiety and depression reported in 50.4 to 60.0% and 30.4 to 36.1%, during the first and second waves, respectively. During the second wave, the authors reported a burnout syndrome in 45.1% using an overall score [23].

In January 2021, a single center study involving 136 healthcare workers (84 nurses, 52 physicians) in a temporary ICU during the pandemic in Milano Fiera, Lombardy reported 60% burnout syndrome, 53% anxiety (especially in nurses), and 45% depression [24]. In June–July 2020, a cross-sectional study involving 709 healthcare providers from 9 English ICUs reported 40% PTSD, 11% severe anxiety, and 6% severe depression. In May 2020, a cross-sectional study involving 352 Swiss ICU healthcare workers reported 22% PTSD, 46% anxiety, and 46% depression [25].

The present study reports lower prevalences of PTSD, anxiety, and depression than the previous ones performed in the first two waves of the pandemic. Our findings could mean that the impact of COVID-19 pandemic has been blunted overtime. Indeed, the present findings are close to those observed at baseline prior to the COVID-19 pandemic [16, 23, 26]. Another explanation could be related to different cultures, different impact of the pandemic and policies on restriction, lockdown, and vaccine strategies in Hong Kong Australia, and France [27–29].

The present study also reported that ICU staff workers in contact with COVID-19 patients are at lower risk of psychological consequences than those not in charge of these patients. This paradoxical phenomenon has been regularly reported in previous studies [14]. Indeed, being far from the patients with no information and education about the disease could lead to fear, anxiety, stress, and other psychological consequences. The absence of information about local progression of the pandemic was also associated with BOS in its 3 dimensions (emotional exhaustion, depersonalization, and loss of personal accomplishment).

In contrast to the previous studies, a quantitative approach to BOS was performed. A threshold of MBI is classically used for diagnosing BOS. However, this dichotomous analysis has been challenged because MBI aggregate 3 different and independent part of the diagnosis. In 2016,

the cut-off scores were removed by the MBI Manual 4th edition because they have no diagnostic validity [30]. Even with this difference, the present study reported similar associated factors with the 3 different parts of BOS (lack of information about local progression of the pandemic and lack of theoretical or practical training on COVID-19 patient management). The present study highlighted several factors associated with PTSD, anxiety, depression, and symptoms of BOS. Moreover, it involved ICUs from different continents. Hong Kong was firstly impacted by the pandemic. France was also severely impacted by the first two waves with some ICU overwhelming episodes. Australia and particularly Queensland closed their borders and had limited transmission and cases in the early stages. Finally, the courses of vaccination covert were different according to the general health strategy against the COVID-19 pandemic. These differences could partly explain the heterogeneous findings of the present study.

We must acknowledge some limitations. First, the participation rate was only 16%, which is consistent with cross-sectional surveys. We did not send personal reminders to respect responder anonymity. Another reason may be the timing of our study (after the third wave, February–July 2021) that was perhaps too far from the start of the pandemic with participant weariness leading to a low response rate. The present study, therefore, likely reported the chronic states of stress, anxiety, depression, and BOS in ICU staff. Second, the cross-sectional survey design only led to isolating associated factors with PTSD, anxiety, depression, and BOS. For isolating risk factors of these psychological disorders, cohort or case–control designs might have been more appropriate. Third, the sample of the present study was not well balanced with a preponderance of French participation. Fourth, non-care giving staff was also underrepresented in this study. Finally, it is well known that the demands of working in ICUs could lead to psychological disorders such as PTSD, anxiety, depression and BOS. As no baseline assessment of these disorders was conducted before the pandemic, we cannot rule out the fact the present study reported only the baseline psychological state [26].

## Conclusion

Our findings confirmed that ICU staff workers continue to suffer from psychological disorders. Even if some factors are linked to the COVID-19 pandemic (fear of managing COVID-19 patients), the lack of theoretical and practical training in the management of COVID-19 patients as well as the lack of information on the current status of the pandemic within the ICU were associated with a higher prevalence of PTSD, anxiety, depression, and BOS. These findings suggest the importance of good communication amongst staff in the ICU for staff wellbeing.

### Supplementary Information

**Additional file 1: Table S1.** Pearson correlation coefficients (between all scales). **Table S2.** Dispersion and position parameters associated with the assessment scales for psychological disorders.

## Data Availability

Nimes University Hospital, BESPIM Department.
