# Supplementary material for: Occurrences of post-traumatic stress disorder, anxiety, depression, and burnout syndrome in ICU staff workers after two-year of the COVID-19 pandemic: the international PSY-CO in ICU study
Source: Ann Gen Psychiatry. 2024 Jan 3;23:3. doi: 10.1186/s12991-023-00488-5 (PMC10765831; doi:10.1186/s12991-023-00488-5)
Supplement: Supplementary file 1 — Additional file 1: Table S1. Pearson correlation coefficients (between all scales). Table S2. Dispersion and position parameters associated with the assessment scales for psychological disorders. [file 12991_2023_488_MOESM1_ESM.docx]

| **Table S1. Pearson correlation coefficients (between all scales)** | | | | | | |
| --- | --- | --- | --- | --- | --- | --- |
|  | **PCL-5 score (95% CI)** | **HADS Anxiety score (95% CI)** | **HADS Depression score (95% CI)** | **MBI Emotional Exhaustion score (95% CI)** | **MBI Depersonalization score (95% CI)** | **MBI Personal Accomplishment score (95% CI)** |
| **PCL-5** | **1** | 0.73 (0.69 – 0.77) | 0.73 (0.69 – 0.77) | 0.67 (0.62 – 0.71) | 0.40 (0.32 – 0.47) | - 0.30 (- 0.37 – - 0.21) |
| **HADS Anxiety** |  | **1** | 0.65 (0.60 – 0.70) | 0.59 (0.53 – 0.64) | 0.29 (0.21 – 0.36) | - 0.29 (- 0.37 – - 0.21) |
| **HADS Depression** |  |  | **1** | 0.62 (0.56 – 0.67) | 0.35 (0.27 – 0.42) | - 0.40 (- 0.47 – - 0.33) |
| **MBI Emotional Exhaustion** |  |  |  | **1** | 0.57 (0.51 – 0.63) | - 0.23 (- 0.31 – - 0.15) |
| **MBI Depersonalization** |  |  |  |  | **1** | - 0.20 (- 0.29 – - 0.12) |
| **MBI Personal Accomplishment** |  |  |  |  |  | 1 |

| **Table S2. Dispersion and position parameters associated with the assessment scales for psychological disorders** | | | | | | | | | |
| --- | --- | --- | --- | --- | --- | --- | --- | --- | --- |
| **Evaluation scale** | | **N^*^** | **Missing data** | **Mean (SD)^ƚƚ^** | **Median (IQR)^ǂǂ^** | **Lower quartile** | **Upper quartile** | **Minimum** | **Maximum** |
| **PCL-5 (Post-Traumatic Stress Disorder)** | | 585 | 0 | 17.2 (14.6) | 14.0 (19.0) | 6.0 | 25.0 | 0 | 78.0 |
| **HADS** | **Anxiety** | 570 |  | 7.1 (4.0) | 7.0 (6.0) | 4.0 | 10.0 | 0 | 19.0 |
|  | **Depression** |  |  | 5.0 (3.8) | 4.0 (6.0) | 2.0 | 8.0 | 0 | 20.0 |
| **MBI** | **Emotional Exhaustion** | 525 |  | 23.5 (13.7) | 22.0 (23.0) | 12.0 | 35.0 | 0 | 54.0 |
|  | **Depersonalization** |  |  | 9.1 (7.0) | 8.0 (11.0) | 3.0 | 14.0 | 0 | 29.0 |
|  | **Personal Accomplishment** |  |  | 35.3 (7.9) | 36.0 (11.0) | 30.0 | 41.0 | 11.0 | 48.0 |
| **^*^**Number of observations  **^ƚƚ^** Standard deviation  **^ǂǂ^** Interquartile range | | | | | | | | | |
